# Management of Hepatocellular Carcinoma Recurrence after Liver Transplantation

**DOI:** 10.3390/cancers13194882

**Published:** 2021-09-29

**Authors:** Filippo Pelizzaro, Martina Gambato, Enrico Gringeri, Alessandro Vitale, Umberto Cillo, Fabio Farinati, Patrizia Burra, Francesco Paolo Russo

**Affiliations:** 1 Gastroenterology Unit, Department of Surgery, Oncology and Gastroenterology, University of Padova, 35128 Padova, Italy; filippo.pelizzaro@unipd.it (F.P.); martina.gambato@gmail.com (M.G.); fabio.farinati@unipd.it (F.F.); burra@unipd.it (P.B.); 2 Multivisceral Transplant Unit, Department of Surgery, Oncology and Gastroenterology, University of Padova, 35128 Padova, Italy; 3 Hepatobiliary Surgery and Liver Transplantation Unit, Department of Surgery, Oncology and Gastroenterology, University of Padova, 35128 Padova, Italy; enrico.gringeri@unipd.it (E.G.); alessandro.vitale@unipd.it (A.V.); cillo@unipd.it (U.C.)

**Keywords:** liver transplantation, hepatocellular carcinoma, recurrence, surveillance, immunosuppression, treatment

## Abstract

**Simple Summary:**

Hepatocellular carcinoma (HCC) is an increasingly important indication for liver transplantation (LT) worldwide. However, LT in the setting of liver cancer is burdened by the risk of tumor recurrence. The prognosis of patients with post-LT HCC recurrence is still very poor and several areas of uncertainty remain in the management of these patients. In this paper, we provide a comprehensive evaluation of available evidence regarding the management of HCC recurrence after LT, starting from the pre- and post-transplant stratification criteria and encompassing post-LT surveillance, preventive strategies and treatment. Much work has been done in the last several years but further effort is still needed in order to improve the outcome of these patients.

**Abstract:**

Recurrence of hepatocellular carcinoma (HCC) after liver transplantation (LT), occurring in 10–15% of cases, is a major concern. A lot of work has been done in order to refine the selection of LT candidates with HCC and to improve the outcome of patients with recurrence. Despite this, the prognosis of these patients remains poor, partly due to the several areas of uncertainty in their management. Even if surveillance for HCC recurrence is crucial for early detection, there is currently no evidence to support a specific and cost-effective post-LT surveillance strategy. Concerning preventive measures, consensus on the best immunosuppressive drugs has not been reached and not enough data to support adjuvant therapy are present. Several therapeutic approaches (surgical, locoregional and systemic treatments) are available in case of recurrence, but there are still few data in the post-LT setting. Moreover, the use of immune checkpoint inhibitors is controversial in transplant recipients considered the risk of rejection. In this paper, the available evidence on the management of HCC recurrence after LT is comprehensively reviewed, considering pre- and post-transplant risk stratification, post-transplant surveillance, preventive strategies and treatment options.

## 1. Introduction

The incidence of hepatocellular carcinoma (HCC) is continuously increasing worldwide, and the prognosis of this tumor still remains very poor, ranking as the second leading cause of cancer-related years of life lost globally [1,2]. Among the several therapies available for HCC patients, liver transplantation (LT) provides the highest survival benefit [3], and in the continuous effort to improve the prognosis of these patients it is not surprising that the rate of transplantation for HCC is progressively increasing. In 2015, HCC, irrespective of underlying etiology, was the most common indication for LT (27% of liver transplants) and placement on waitlists (24% of waitlist additions) in the United States [4].

A milestone in the history of transplantation for HCC was the demonstration in 1996, by Mazzaferro et al., that LT was an effective therapy for those patients having a tumor burden within the so-called Milano criteria (single lesion ≤5 cm or up to three lesions all ≤3 cm, without vascular involvement or extrahepatic spread) [5]. Over time, several extended criteria for the selection of LT candidates have been proposed with the goal of maintaining an acceptable risk of HCC recurrence and survival [6,7,8,9,10,11,12,13,14]. Indeed, even if survival benefit of LT as compared to alternative therapies has been demonstrated regardless of tumor burden (provided that macroscopic vascular invasion and extrahepatic spread are absent) [15], in the selection of the optimal candidates not only transplant benefit, but also transplant utility (i.e., the selection of patients with low post-LT recurrence risk) should be considered [16]. It is necessary to identify the limits beyond which LT becomes futile due to an unacceptable risk of recurrence and low survival. Indeed, as a matter of fact, HCC recurrence is one of the most relevant negative predictor of post-LT survival. In the study by Mazzaferro et al., the 4-year recurrence rate after LT was 8% [5]. Other studies, focusing on patients within Milano criteria as evaluated before transplant, demonstrated post-LT recurrence in 10–16% of patients [17,18,19]. A mean rate of HCC recurrence of 16% was demonstrated in a systematic review including 61 studies, with half of the patients classified as beyond the Milano criteria at the explant pathology evaluation [20]. Considering that many centers worldwide transplant patients beyond the Milano criteria, it can be speculated that the magnitude of HCC recurrence is greater than that estimated by these studies.

Considered the prognostic relevance of tumor recurrence after LT and the lack of guidance for the management of transplanted patients to maximize their outcomes, in this paper we provide a comprehensive review of the available evidence on HCC recurrence after liver transplantation, encompassing pre- and post-transplant risk stratification, post-transplant surveillance, preventive strategies and treatment options.

## 2. Risk Factors and Scoring Systems for Post-Transplant HCC Recurrence and Survival

Many prognostic scoring systems, developed with the aim of accurately predicting the risk of HCC recurrence and survival after LT, have been proposed. These pre-LT and post-LT prognostic models include several tumor-related characteristics and risk factors that have been identified as predictors of patient outcomes after transplantation.

### 2.1. Pre-Transplant Prognostic Models and Selection Criteria

In the process of selecting LT candidates, the widening of transplantability boundaries and the minimization of the risk of recurrence should be balanced. A minimum survival threshold must be reached to justify expansion, while not harming non-HCC patients on the waiting list [16,21]. The available pre-transplant selection models are shown in Table 1.

Traditionally, selection criteria relied only on the evaluation of tumor burden. Among these pre-transplant prognostic scoring systems, the Milano criteria [5] are the benchmark for LT candidates’ selection and the comparator for other proposed criteria. Other criteria relying on size/number alone, such as UCSF criteria (single nodule ≤6.5 cm or 2–3 nodules ≤4.5 cm and total tumor diameter ≤8 cm) [6,7] and Up-to-7 criteria (the sum of the diameter (cm) of the largest tumor and the number of nodules must not exceed the limit of 7) [10], even expanding the boundaries of transplantability, have demonstrated comparable survival results. However, increasing experience demonstrates a concept that is nowadays widely accepted: the further outside the Milano criteria, the greater the risk of recurrence [6,7,10,14]. Interestingly, for patients with tumor burden beyond the Milano criteria, successful downstaging to within these boundaries is associated with a rate of HCC recurrence and survival comparable to those meeting the Milano criteria without downstaging [22]. Thus far, considering that patients who progress despite locoregional therapies exhibit worse post-LT outcomes [23,24,25], the strategy to consider the response to locoregional therapies as a marker of favorable tumor biology has gained broader acceptance as an additional risk stratification tool [26,27].

Number of lesions and tumor size, while representing the disease burden at the time of transplant, do not necessarily reflect the biology and aggressiveness of the tumor. Serum biomarkers, considered both as static and dynamic variables, may provide accurate information on tumor biology and thus on post-transplant recurrence risk. Among these biomarkers, the majority of data are for alpha-fetoprotein (AFP), which is demonstrated to be a useful predictor of the risk of drop-out while on the waiting list for LT, the risk of tumor recurrence after LT and overall survival (OS) (Table 2). A direct correlation between AFP levels before transplantation and post-LT mortality has been demonstrated, with progressively worsening outcomes as levels increase, starting from values as low as 16–20 ng/mL [28,29,30]. Interestingly, the prediction of 5-year disease-free survival rate was superior for the combined assessment of pre-transplant AFP (with a cut-off of 200 ng/mL) and ^18^F-fluorodeoxyglucose positron emission tomography in comparison to the Milano criteria [31]. AFP as a dynamic factor can also provide valuable information on the biology of the tumor. The increase of AFP levels before LT have been associated with HCC recurrence and worse survival after transplantation, but the threshold of the AFP slope is variable among different studies (>7.5 ng/mL, >15 ng/mL or >50 ng/mL per month) [25,32,33,34,35]. By contrast, successful reduction of AFP levels after locoregional therapy is predictive of better prognosis [36,37,38,39,40]. In addition to AFP, other serum biomarkers, such as the neutrophils-to-lymphocytes ratio (NLR), AFP-L3 and des-γ-carboxyprothrombin (DCP), resulted to be surrogate markers of tumor biology and have been considered for inclusion in pre-transplant prognostic models [41,42,43,44]. The majority of recently developed selection criteria, in order to improve the prediction of post-LT outcomes, include the evaluation of serum biomarkers [11,12,14,38,44,45,46,47,48]. In particular, since most of the data are on AFP as a powerful prognostic marker, its evaluation has been incorporated in almost every recently developed prognostic model (Table 1). However, no consensus has been reached on AFP threshold as an exclusion criteria and various cut-offs have been proposed, such as 100 ng/mL [11,49,50], 200 ng/mL [31], 400 ng/mL [12,47,51,52,53] and 1000 ng/mL [11,22,28,54,55]. The recently developed metroticket 2.0 model considers AFP as a continuous variable, and its variations along with tumor morphology can be used as an accurate predictor of tumor-related death after liver transplantation [14]. It has also been demonstrated that metroticket 2.0 can be improved in its predictive ability by incorporating information relating to the radiological responses of patients (mRECIST) to neoadjuvant therapies [56]. Another very recent study investigated the effect of evaluating tumor burden with Liver Imaging Reporting and Data System (LI-RADS) classification on prognostic accuracy of metroticket 2.0 [57]. Although single-center and retrospective, this study demonstrated that not considering LR-3 and LR-4 nodules (with intermediate probability of being HCC) in metroticket 2.0 resulted in a significant drop of its accuracy (c-index 0.72 and 0.60, respectively; *p* = 0.009). By contrast, the inclusion of all vital nodules (LR3, LR4, LR5 and LR-TR-V) raised metroticket 2.0 c-index to 0.65, which is not significantly different from its original performance (*p* = 0.08). Waiting for multicenter prospective studies, these data suggest that every intermediate-to-high risk nodule according to LI-RADS protocol should be considered when applying the metroticket 2.0 calculator in order to achieve an appropriate performance.

Since liver biopsy is not mandatory to achieve HCC diagnosis and it is not routinely performed, the ability to integrate in selection criteria and in pre-transplant prognostic models information about tumor differentiation or genomic risk stratification is limited. In a study published in 2004, post-LT outcomes were evaluated in 33 patients transplanted for HCC irrespective of tumor burden after excluding the biologically most aggressive tumors according to grade (i.e., grade III and IV) at pre-operative liver biopsy [8]. On explant pathology 38% of patients were beyond the Milano criteria, the 5-year survival rate was 75% and the recurrence-free survival was 75%. The favorable results achieved with the use of these selection criteria were subsequently confirmed in a prospective study by the same research group [9]. Tumor grading as a pre-LT selection tool has also been included in the extended Toronto criteria, in which nodule size and number are not contraindications to LT as instead the presence of poorly differentiated tumors, cancer-related symptoms, macrovascular invasion and extrahepatic spread [13]. However, relying on liver biopsy to evaluate tumor biology might be burdened by some issues, such as sampling bias and intratumoral heterogeneity [58,59]. According to one study, the agreement of cases identified as poorly differentiated by pre-operative needle biopsy and explant pathology was poor (15% vs. 28%, respectively) [60]. In another recent series, percutaneous liver biopsy demonstrated not only poor concordance with final explant pathology, but also low sensitivity and positive predictive value (29% and 35%, respectively) in identifying poorly differentiated tumors [61]. Therefore, there are still some concerns on liberalizing tumor burden and relying exclusively on pre-LT histological assessment of tumor grade.

### 2.2. Post-Transplant Prognostic Models

Post-transplant prognostic scoring systems have been developed in order to provide reliable evaluations of the recurrence risk, also including information available after LT (i.e., explant pathology) (Table 3). Among the well-established explant features with prognostic relevance, microvascular invasion is strongly associated with HCC recurrence and reduced survival [65,66]. In addition, poor tumor differentiation has also been identified as a relevant risk factor for recurrence in several studies [67,68,69].

A pure pathologic risk score based on four tumor characteristics on explant liver (microvascular invasion, tumor size ≥3 cm, satellitosis and giant/bizarre cells >25% visible at low power) proved to be able to predict recurrence with an AUC of 0.8 (sensitivity 80% and specificity 79% at a cut-off value ≥3.5) [65,70]. Decaens et al. developed a score including number/size of tumors and tumor differentiation (Edmonson–Steiner grade) [71]. The authors, in their paper, claimed that a cut-off of 4 was able to discriminate groups at low versus high risk of recurrence (5-year tumor-free survival of 82.8% vs. 50.0%, respectively; *p* = 0.0003), but they did not provide further indications to guide management. Subsequently, a prognostic nomogram accounting for several variables (tumor grade, vascular invasion, within Milano/downstaged to Milano vs. outside Milano, maximal radiological tumor diameter, AFP, NLR and total cholesterol) was published by Agopian et al. [42]. Even this score, although providing predictions of 1-, 3- and 5-year recurrence risk with a high accuracy (C statistic of 0.85, 95% CI 0.82–0.89), is not useful for identifying categories of patients needing a tighter surveillance or adjuvant treatment. On the contrary, the post-transplant scoring system proposed by Halazun et al. (post-MORAL) [45] based on tumor grading, vascular invasion, larger tumors >3 cm and number of tumors >3 on explant, accurately stratifies patients in four groups according to recurrence risk (C statistic 0.88, 95% CI 0.83–0.93). The accuracy in recurrence risk prediction was also further improved (C statistic 0.91, 95% CI 0.87–0.95) by proposing the combo-MORAL score, which combined the pre-transplant (pre-MORAL) and the post-transplant (post-MORAL) models. Finally, in the RETREAT score AFP, vascular invasion and sum of the largest viable tumor diameter (cm) and number of viable tumors on explanted liver were combined to obtain a scoring risk from 0 to >5 [29]. In the external validation using a cohort from the UNOS database, this score confirmed to be able to adequately divide patients according to the risk of recurrence (post-LT HCC recurrence probability within 3 years progressively increased from 1.6% to 29% with RETREAT score going from 0 to ≥5) [30]. In addition, patients with a high RETREAT score (≥4) had a median recurrence recurrence-free time significantly shorter compared to patients with a score of 0–1 (10.9 months vs. 14.0 months; *p* = 0.03).

Post-transplant scoring systems not only help the identification of patients with high risk of recurrence, but may also be useful in the development of standardized surveillance protocols and in the identification of patients that should be considered for clinical trial using adjuvant therapy given their particularly high risk of HCC recurrence. Concerning this, it is worth to consider that, thus far, only the Parfitt et al., Decaens et al. and RETREAT scores have been externally and independently validated.

## 3. Post-Transplant Surveillance for HCC Recurrence

Considered the known risk of recurrence of HCC after LT, patients deserve periodical monitoring for cancer reappearance. Surveillance exerts its benefit on prognosis through the identification of early recurrence, allowing in turn the applicability of potentially curative treatments [72].

In order to be more cost-effective, surveillance after LT should be tailored according to the known pattern of recurrence (i.e., in a time frame covering the majority of recurrences, using imaging modalities that evaluate anatomical sites where recurrences are known to occur) (Table 4). Timing of HCC recurrence is variable, but in the majority of cases it occurs 2–3 years after LT [19,72,73,74,75,76]. Early recurrence (defined as <1 year after LT) is associated with a significantly worse prognosis [72,77], while on the contrary later recurrence is a predictor of better outcomes [71,78,79]. Although in rare cases, also HCC recurrence after 5 years from LT have been described [74,77,80]. Considered the timing of HCC recurrence reported in the literature, it has been suggested that post-LT surveillance should be more intense for 2–3 years after LT and should be maintained at least for 5 years in high-risk patients. HCC can reappear both at intrahepatic (in the graft) and at extrahepatic level, and can involve a single site or multiple sites [19,29,72,73,74,75,76,77,78,81]. The most common extrahepatic sites of recurrence are lung and bones. However, adrenal glands, peritoneum, soft tissue (e.g., on the needle track of percutaneous liver biopsy) and central nervous system may also be involved [17,29,73,74,81].

Although clinical characteristics of HCC recurrence have been thoroughly investigated, there is still a substantial lack of data about the optimal tests and their schedule. In particular, no trials evaluating the effectiveness of surveillance protocols and their impact on post-LT prognosis are available. While it remains to be conclusively proven whether post-LT surveillance improves HCC-related outcomes, some data about the utility of keeping monitored these patients for the development of recurrence have been published. Lee et al. [82] demonstrated that the cumulative exposure to surveillance (CETS), defined as the cumulative sum of all the protected intervals that each screening test provides, was independently associated with a reduced risk of post-recurrence mortality (adjusted hazard ratio [HR] = 0.94, 95% CI 0.91–0.98), with an upper limit provided by more frequent surveillance. It was also showed that the highest probability for curative post-recurrence treatment was provided with 252 days of CETS within the first 24 months [82].

Despite the expected benefits of surveillance, no standardized and commonly accepted protocols are currently present, mainly because there are no clinical trials’ data to guide recommendations on the best way to monitor these patients [83]. A recent national survey in the United States revealed that, while the majority of the centers reported to have a routine imaging protocol for post-LT surveillance, a considerable heterogeneity in frequency and duration of such surveillance is present [84]. Moreover, only about 50% of centers of those using recurrence risk stratification scores modify surveillance schedule according to the expected risk of HCC recurrence [84].

Standard surveillance strategies involve cross sectional imaging of the abdomen (either contrast-enhanced computed tomography [CT] or magnetic resonance imaging [MRI]), and imaging for lung metastasis with non-contrast enhanced chest CT. Bones also may be a common site of recurrence, but bone scans are not routinely recommended due to their low sensitivity [85]. Instead, specific investigations should be performed on demand in patients presenting with an elevation of alkaline phosphatase or with pain at the site of bone metastasis [85]. As far as surveillance interval is concerned, it is generally recommended to repeat imaging investigation (abdomen contrast-enhanced CT or MRI, chest CT) every 6 months [85]. This seems reasonable considering that the few available data demonstrated similar recurrence-free survival for patients undergoing 3- or 6-month post-LT surveillance with CT [86]. However, prospective validation of the semiannual surveillance schedule is still needed.

Due to its low cost and easy determination, AFP levels are usually evaluated and their determination every 6 months at least for 5 years after LT is recommended [64,87]. No other biomarkers (e.g., AFP-L3%, DCP) are commonly monitored in routine post-LT surveillance programs due to the lack of data.

Considered that the majority of patients recur 2–3 years after LT, a minimal duration of surveillance of at least 3 years has been proposed [83,85]. Many programs continue surveillance upon 5 years post-LT and not beyond this point, considered the low likelihood of recurrence thereafter. However, it is again not possible to make strong recommendations on the best or most cost-effective surveillance length due to the lack of data.

Since some patients may deserve more enhanced monitoring than others based on post-transplant recurrence risk, an interesting approach could be the individualization of surveillance schedules. Mehta et al. proposed to stratify patients in groups according to their risk of HCC recurrence evaluated with the RETEAT score and tailor surveillance strategies accordingly [30]. Patients with a RETREAT score of 0 should not undergo surveillance, considering their low risk of recurrence (5-year recurrence rate of 3%). Progressively enhancing surveillance schedules have been proposed with increasing values of RETREAT score: every 6 months for 2 years after LT in patients with a RETREAT score of 1–3; every 6 months for 5 years after LT in patients with RETREAT score of 4; every 3–4 months in the first 2 years post-LT followed by a 6-month surveillance interval from the second to the fifth year after LT in those with RETREAT score ≥5 [30,83]. Nevertheless, although this approach might be clinically useful, it still needs to be prospectively validated and its cost-effectiveness should be proved.

## 4. Management of HCC Recurrence

### 4.1. Immunosuppression

Considering the major defense against cancer provided by the immune system, the role of immunosuppressive regimes in influencing the risk of HCC recurrence after LT has been thoroughly studied. Calcineurin inhibitors (CNIs), tacrolimus and cyclosporine, are the cornerstone drugs of immunosuppressive therapy, but they can create a permissive environment for the growth of cancer cells due to the impairment of organ recipient’s immune-surveillance system. Data derived from in vitro studies and animal models demonstrated that CNIs promote tumor growth and cancer progression [93,94]. Early report showed that growth rate (tumor doubling time) of the recurrent HCC in patients receiving immunosuppression with cyclosporine and steroids is greater than that of those with recurrence after liver resection not receiving immunosuppression [95]. Subsequent retrospective studies confirmed that CNI therapy is associated with an increased risk of tumor recurrence, especially if high trough blood levels of these drugs are maintained in the early post-transplant period [96,97,98]. Moreover, a dose-dependent direct association with an increased risk of post-transplant HCC recurrence has been demonstrated for CNIs [98].

In order to overcome this limitation, immunosuppressive regimens based on mammalian target of rapamycin (mTOR) inhibitors, sirolimus and everolimus, have been developed. mTOR has a central role in regulating many fundamental cell processes, and its deregulation has been implicated in the progression of human cancers, including HCC [99,100,101,102,103]. A great number of mutations found in HCC involve abnormal upregulation of mTOR expression [104], and tumors with increased signaling of mTOR have been recently identified as a subset of aggressive cancers [105]. In contrast to CNIs, mTOR inhibitors have been shown to inhibit HCC growth in vitro and in animal models [103,106].

Data from retrospective studies and metanalyses suggest that, compared to CNIs, the use of sirolimus reduced the risk of post-LT HCC recurrence [107,108,109,110,111,112,113,114,115]. In their large study, Toso et al. analyzed the data collected in the Scientific Registry of Transplant Recipients (SRTR) database and found an improvement in survival for patients managed with a sirolimus-based maintenance therapy (HR = 0.53, 95% CI 0.31–0.92) [110]. By contrast, while retrieving the data from the same database (SRTR), Yanik et al. found no differences in OS between sirolimus and no-sirolimus treated patients, even though a reduction in cancer-specific mortality and HCC recurrence was demonstrated [111]. One important difference between these latter two studies was the method of ascertaining the sirolimus usage. While Toso et al. relied on immunosuppression data at the time of hospital discharge, Yanik et al. linked the SRTR database with pharmacy claims [110,111]. A systematic review including 3666 HCC liver transplant recipients showed that CNI immunosuppressive regimens were associated with an increased risk of HCC recurrence compared to mTOR inhibitor-containing regimens (13.8% vs. 8.0%, *p* < 0.001) [114]. In order to achieve a definite answer on the beneficial effect of sirolimus, a multicenter phase 3 randomized controlled trial, the SiLVER trial, was designed [116]. After 6 weeks of non-mTOR inhibitor containing regimen, patients were randomized to continue without mTOR inhibitor versus the incorporation of sirolimus in the immunosuppressive therapy. Significant benefits of the mTOR inhibitor were only observed in patients within Milano criteria (i.e., low-risk patients). Moreover, while improved RFS and OS were observed in sirolimus containing group in the first years after LT, these differences were no longer sustained thereafter [116]. A subsequent post-hoc analysis demonstrated a significant increase of OS in some subgroups, namely younger patients (<60 years), those using sirolimus for ≥3 months and patients with active tumors at the time of transplantation (AFP ≥10 ng/mL) [117].

Whether same findings would be observed with everolimus is still unknown. Everolimus treatment proved not to be associated with tumor recurrence, neither at multivariate Cox regression nor in a competing-risk analysis for tumor recurrence death, in a study including 192 HCC patients undergoing liver transplantation [118]. Blood trough levels seem to be associated with HCC recurrence rate, and were found to be significantly higher in patients with recurrence [119]. On the contrary, a monocentric retrospective study demonstrated that patients treated with everolimus in combination with CNIs had significantly better time to recurrence and OS compared to those receiving CNIs alone [120]. Very recently, the 24-month results from the pooled analysis of two randomized controlled trials, comparing everolimus with reduced tacrolimus versus tacrolimus alone, showed that HCC recurrence was numerically lower, although not statistically significant, with everolimus use (5.9% vs. 23.1%, *p* = 0.22) in patients transplanted for HCC beyond the Milano criteria, while comparable in patients within the Milano criteria (2.9% vs. 2.1%, *p* = 0.1) [121]. A clinical trial comparing tacrolimus and everolimus versus tacrolimus and mycophenolate mofetil in patients transplanted for HCC is currently ongoing and results are awaited [122].

The optimal immunosuppression strategy to minimize the frequency of HCC recurrence and improve the survival, including the precise role of mTOR inhibitors, has not yet been determined [123]. Moreover, in clinical practice, side effects profile and comorbidities including kidney dysfunction must also be considered in the choice of the optimal immunosuppression strategy for a specific patient. However, while waiting for further well-designed multicenter studies delineating the use of mTOR inhibitors, an attempt in implementing strategies to reduce CNIs in order to limit the impact of their exposure on cancer recurrence is reasonable [124].

### 4.2. Pre-Emptive Therapy

HCC is commonly characterized as a chemoresistant tumor. Despite several chemotherapeutic schemes having been proposed [125,126,127,128,129,130,131,132,133], evidence supporting the use of adjuvant systemic chemotherapy in preventing post-LT recurrence is lacking and it is currently not recommended. Some promising preliminary results have been obtained with the use of licartin, a novel drug in which metuximab (a monoclonal antibody targeting HAb18G/CD147, an antigen overexpressed in HCC) is conjugated to the radioisotope [131]. The administration of adjuvant licartin proved to significantly reduce HCC recurrence rate as compared to controls (27% vs. 57%, *p* = 0.017) in a randomized study enrolling 60 transplanted patients with HCC beyond the Milano criteria [131].

Initially, adjuvant treatment with sorafenib appeared to be potentially useful. In a phase I trial involving 14 patients, at a maximum tolerable dose of 200 mg BID, sorafenib proved to be safe and probably effective, with only one death and four recurrences after a median follow-up of almost 32 months [134]. However, the usefulness of sorafenib in the adjuvant setting post-LT has been scaled back subsequently, even though the data derive from small single-center studies and case series [135,136,137,138]. In one of the largest available studies, Satapathy et al. compared treatment with pre-emptive sorafenib (*n* = 25) versus standard of care (*n* = 20) in patients with advanced HCC at explant pathology, finding no significant differences in recurrence-free and overall survival between the two groups [138]. These data on the lack of benefit for adjuvant sorafenib are concordant with what was already demonstrated in the STORM trial for resected and ablated patients [139]. Only care reports are available on the role of lenvatinib as an adjuvant therapy after LT [140], and LT recipients are excluded from the currently ongoing trials examining the effect of immune checkpoint inhibitors in patients with high risk of recurrence after resection and ablation.

## 5. Treatment of Post-Transplant HCC Recurrence

Considering that strategies for prevention are limited, the management of HCC recurrence is focused on treatment, which should be prompt and possibly curative. However, even if several therapeutic approaches are currently available, patients with post-LT HCC recurrence are burdened with a severe prognosis, particularly in the case of early recurrence.

### 5.1. Surgical Resection

Patients who recur after LT had a worse prognosis compared to those whit tumor reappearance after surgical resection (median survival after recurrence of 10–13 months compared to nearly two years, respectively) [83,141,142,143,144]. The necessary immunosuppressive state after transplantation, which inhibits patient’s immunosurveillance system in its ability to clear micrometastatic disease and slow tumor growth, may contribute to explaining this difference [97]. Nevertheless, patients with recurrent HCC may have a clear benefit when treated with therapies with curative intent [19,72,73,75,89,141]. Sapisochin et al. retrospectively analyzed 121 patents with HCC recurrence after LT, finding that not being amenable to resection or ablation was an independent predictor of poor prognosis (HR = 4.7, 95% CI 2.7–8.3) [72]. In a single-center retrospective study evaluating 106 patients with HCC recurrence after LT, it was demonstrated that patients treated with surgery alone (23.3%) had a significantly longer survival (27.8 months) compared to those receiving both surgical and nonsurgical therapy (10.6 months), and nonsurgical therapy alone (3.7 months) [141]. Similarly, an Italian multicentric study reported a significantly better 4-year survival rate in patients treated with surgical resection for intra- and extra-hepatic recurrence compared to those with unresectable disease (57% vs. 14%, *p* = 0.02) [89]. This was also confirmed by another recent study showing that the survival of 22/70 patients treated with surgical resection after HCC recurrence (2 intrahepatic and 20 extrahepatic) was significantly longer compared to that of non-resected patients (35 vs. 15 months, *p* < 0.001) [75]. Better results have been obtained by Kornberg et al., with a median survival after tumor relapse of 65 months in 7/16 HCC recurrent patients amenable for surgical therapy compared to 5 months in those not suitable for surgery (*p* = 0.01) [73]. In a small retrospective study, Valdivieso et al. reported the outcomes of 11 recurrent HCC patients undergoing surgical resection [19]: 4/8 patients with R-0 resection and all the three patients with R-1 resection eventually developed second recurrence, but OS from recurrence was still significantly longer in the R-0 group compared to patients not treated surgically (33.2 vs. 11.9 months, *p* = 0.006; 5-year survival rate of 27% in R-0 patients vs. 0% at 3 years in the other cohort). In patients with multiple recurrences, some benefits have also been shown for repeated resections, probably reflecting less-aggressive tumor biology [75]. In addition, for patients with oligometastatic disease, the resection of isolated extrahepatic recurrent HCC in regional lymph nodes, adrenal glands and lungs has shown favorable results [145,146,147].

### 5.2. Locoregional Therapies

Data on liver-directed locoregional therapies for the management of HCC recurrence after LT are largely lacking, with only some small case series currently available. In a retrospective study, Huang et al. compared 15 patients with post-LT HCC recurrence treated surgically with 11 patients treated with radiofrequency ablation (RFA), showing no differences in the 5-year OS (35% vs. 28%, *p* = 0.88) and a worse disease-free survival, although not statistically significant, in the RFA group (16% vs. 0%; *p* = 0.75) [148]. Microwave ablation (MWA) also may be useful in treating HCC recurrence after LT [149]. Zhai et al. evaluated safety and efficacy of MWA in a series of 11 LT recipients with intrahepatic recurrent HCC, finding the procedure was well tolerated and only three cases had tumor progression after treatment (1–7 months) [150]. However, survival rate at 2 years was 15.3% and the mean survival time was 17.3 months.

The efficacy of conventional transarterial chemoembolization (TACE) in 28 patients with recurrent HCC after living donor LT was evaluated by Ko et al. [151]. A reduction in tumor size ≥25% was observed in 19/28 patients (67.9%), but intrahepatic or extrahepatic metastasis was observed in 92.9% of patients in the first 6 months of follow-up after TACE. Moreover, prognosis was very poor, with 1-, 3- and 5-year survival rates resulting 47.9%, 6.0% and 0%, respectively, and a mean survival of 9 months [151]. Similar results were obtained by Zhou et al. who demonstrated a significantly improvement in post-recurrence survival in TACE group, despite the prognosis remaining far from being satisfactory (286 days vs. 85 days, *p* = 0.03) [152].

### 5.3. Systemic Therapies

Since its approval following the positive results of the SHARP and the Asia-Pacific trial [153,154], systemic therapy with sorafenib became the standard of care for advanced stage HCC, and it has also been studied in the management of non-resectable recurrent HCC after LT. However, its safety and effectiveness in this setting has not been evaluated in randomized clinical trials, and available data are limited and restricted to small studies and case series. A single-center retrospective investigation form Italy compared 15 patients treated with sorafenib and 24 receiving best supportive care (BSC) after HCC recurrence, demonstrating a significantly improved recurrence-free survival in the former group (21.3 vs. 11.8 months, HR = 5.2, *p* = 0.0009) [155]. In their metanalysis, Mancuso et al. demonstrated that patients with recurrence receiving sorafenib after LT had a pooled 1-year survival of 63% (ranging from 18% to 90%) [156]. Overall, a median survival of 12 months (range 1.45–21.3) was demonstrated for sorafenib treatment in this setting [155,157,158,159,160,161,162].

Sorafenib use in the after-LT period may be limited by side effects and its safety is a point of concern. More than half of patients with recurrent HCC treated with sorafenib require dose reduction [137,155]. In a recent analysis of the United States cohort of the GIDEON registry, the safety and tolerability of this drug was evaluated in patients with HCC recurrence after LT and after surgical resection [163]. This study demonstrated that most adverse events occurred in the first 4 weeks of treatment and the incidence of toxicities requiring sorafenib discontinuation was similar between the two groups. Therefore, the authors were not able to conclude that sorafenib in post-transplant setting was associated with an increased toxicity compared to patients treated for their primary HCC. However, close monitoring is mandatory, not only during the first weeks of treatment, but also in the case of immunosuppression with mTOR inhibitors since this association demonstrated an increase in the rate of dose reduction and discontinuation due to severe adverse events [162,164,165,166,167].

In patients progressing while on treatment with sorafenib, regorafenib in second-line setting may be an option [168]. It proved to be a safe and tolerable treatment in a preliminary evaluation [169], and very recently a multicenter retrospective cohort study including 81 patients (36 treated with regorafenib and 45 undergoing BSC at sorafenib discontinuation) confirmed these findings [170]. From sorafenib discontinuation, regorafenib was able to provide a significantly longer OS compared to BSC (13.1 vs. 5.5 months, *p* = 0.002) and was independently associated with lower mortality (HR = 0.37, 95% CI 0.16–0.89) [170]. The median OS of the sorafenib/regorafenib sequence (considered from the beginning of sorafenib) was 28.8 months. All regorafenib-treated patients experienced side effects, but adverse events (grade ≥ 3) were severe in 14 patients (38.9%) [170]. Currently, no data except for case reports [171] are available for other recently approved tyrosine kinase inhibitors (lenvatinib [172] and cabozantinib [173]) and monoclonal antibodies (ramucirumab [174]). Although transplanted patients have been excluded from all registration trials, these drugs will likely be adopted soon in real-life series in post-LT settings, as previously done with sorafenib and regorafenib, thus allowing the collection of data on new treatment sequences to adopt in this special population [175].

The use of other systemic chemotherapy agents has been evaluated for the treatment of HCC recurrence after LT, but data are again very limited. Metronomic capecitabine (administered in low doses without breaks) proved to be safe and effective in advanced HCC [176,177,178], and it was evaluated also in 38 patients with post-LT HCC recurrence [179]. The safety profile was similar to that of sorafenib and a significantly increased survival after tumor recurrence was reported compared to patients undergoing BSC (median 22 vs. 7 months; *p* < 0.01).

Very recently, several studies evaluating the role of immunotherapy in HCC have been conducted and more are currently ongoing. ICIs are designed to target programmed cell death protein-1 (PD-1)/programmed death-ligand 1 (PD-L1) or cytotoxic T-lymphocyte-associated antigen 4 (CTLA-4), all fundamental negative regulators of T cell function [180]. These drugs act through the stimulation of an effective antitumor immune response, allowing immune system to recognize and destroy cancer cells. However, the reactivation of the immune system is not only directed against the tumor and can also lead to immune mediated adverse events, similar to autoimmune diseases, as a consequence to the loss of the ability to recognize self from non-self. Following positive results of phase II clinical trials (Checkmate 040 [181] and Keynote 224 [182]), the Food and Drug Administration granted conditional approval the PD-1 inhibitors nivolumab and pembrolizumab in second-line after sorafenib. Unfortunately, recent phase III studies failed to confirm a statistically significant advantage in progression-free and OS for pembrolizumab [183]. No phase III data on nivolumab in second-line are available at this time, but the OS of nivolumab treated patients in first-line was not significantly longer compared to those receiving sorafenib in the Checkmate 459 phase III trial [184]. A significant step forward in the management of advanced HCC and a robust proof of the effectiveness of immunotherapy has been achieved very recently following the positive results of the IMbrave150 trial [185]. More than 10 years after sorafenib approval, the combination of atezolizumab (an anti-PD-L1) and bevacizumab (anti-vascular endothelial growth factor (VEGF) monoclonal antibody) proved to be superior over sorafenib in first line.

Despite the very promising results with immunotherapy, the possibility to safely use ICIs in post-transplant setting remains a relevant issue. In solid organ transplant recipients, PD-1/PD-L1 pathway is fundamental in regulating alloimmunity and transplant tolerance [186]. Therefore, the use of ICIs after transplantation may expose these patients to the risk of allograft rejection and graft loss, with more severe cases that may progress even to death [175,187,188]. Several case reports have been published on the use of drugs targeting CTLA-4 and PD-1/PD-L1 in patients with HCC, melanoma and non-small cell lung cancer after liver, kidney, heart and corneal transplants [188,189,190,191,192,193]. Safety and efficacy of PD-1 inhibitors was reported in a retrospective pilot evaluation of the Mayo Clinic experience in seven patients (five with recurrent HCC and two with melanoma) after LT [190]. In this study, three out of seven patients discontinued the treatment early (two because of graft rejection and one because of multiorgan failure unrelated to therapy). Among the four patients evaluable for response assessment, three patients with HCC recurrence progressed and a complete response without graft rejection was observed in one patient with metastatic melanoma. Literature reports 25–54% graft rejection rates, occurring rapidly after the start of immunotherapy (8–19 days) [190,191,192]. In the case of graft rejection, some effective treatments are available (steroids, mycophenolate mofetil and antithymocyte globulin), but in several cases of patients with recurrent HCC after LT a rapid irreversible liver dysfunction and death after ICIs treatment occurred [188,191]. There are some interesting but very limited data showing that staining for PD-1 expression in liver allograft may be predictive of rejection, with lower risk in patients lacking PD-1 positive lymphocytes [190]. However, considering that published data highlight the detrimental risk of graft loss under immunotherapy and no robust data allowing the prediction and identification of patients at increased risk of graft rejection and loss are available, ICIs should not be recommended in the setting of HCC recurrence after LT to date.

## 6. Conclusions

Although being the most effective therapy available in the treatment of HCC, LT remains burdened in these patients by the risk of tumor recurrence. Although with low incidence, patients who experience recurrence have a dismal prognosis. In the last few decades, several steps forward have been made in the management of post-LT HCC recurrence. However, there is still room for improvement and work needs to be done in order to refine the outcome of these patients.

Careful patient selection and stratification is a crucial point. A lot of pre-transplant prognostic models have been proposed but, in most cases, they lack robust prospective independent validation. An ideal pre-transplant model, useful in improving post-LT outcome, should combine a surrogate of tumor biology with conventional morphologic criteria. It is commonly recognized that monitoring transplanted patients for HCC recurrence after LT is fundamental, but until now there has been no definite evidence of the benefits of surveillance on survival, and the most cost-effective strategy has not been identified. Moreover, work still need to be done in risk stratification, possibly by developing post-LT prognostic models useful in identifying those patients deserving closer monitoring for their higher risk of recurrence. Preventive strategies to reduce the risk of recurrence have not been delineated thus far. Well-conducted large studies will identify the best immunosuppressive regimen and, possibly, adjuvant treatments that will help to improve post-transplant outcomes. For patients experiencing recurrence a large variety of treatments is available and, whenever possible, curative-intent therapies should be delivered. In the last few years, there has been a significant expansion in the systemic therapy options for patients with advanced HCC. However, we have still very few data on the role of tyrosine kinase inhibitors in post-LT setting, since these patients have been excluded from registration trials. There is an urgent need for studies investigating safety and efficacy of these molecules in the treatment of HCC recurrence after transplantation and the best therapeutic approach. An area of extreme interest is immunotherapy which is revolutionizing the treatment of HCC. Treatment with ICIs will likely not be possible in all patients with HCC recurrence after LT because of the risk of allograft rejection and graft loss. Nevertheless, an effort should be made in identifying factors predictive of rejection under ICIs and patients who will likely benefit most from this treatment.

## Figures and Tables

**Table 1:** Proposed pre-transplant selection criteria and scoring systems.

Model Name	Year	Design	Tumor Burden	Biomarker	Other Criteria	Performance
Milano criteria [5]	1996	Prospective single-center study	Single tumor >5 cm or ≤3 tumors ≤ 3 cm	-	No vascular invasion or lymph nodes	5-year OS: 85% 5-year RFS: 92%
UCSF criteria [6,7]	2001	Retrospective evaluation of prosepctively collected data, single center [6]	Single nodule ≤6.5 cm or 2–3 nodules ≤4.5 cm and total tumor diameter ≤8 cm	-	No vascular invasion	5-year OS: 75.2% in [6] 5-year RFS 80.9% in [7]
		Prospective single-center study [7]				
University of Padova selection criteria [8,9]	2004	Retrospective evaluation of prosepctively collected data, single center [8]	Any size or number of tumors	-	No vascular invasion/extrahepatic spread No poorly differentiated tumor (grade III and IV)	5-year OS: 75% 5-year RFS: 92% In [9] 1-, 3- and 5-year ITT survival rates were: 95%, 85% and 79% in Milano out 84%, 69% and 69% in Milano in
		Prospective single-center study [9]				
Seoul criteria [48]	2007	Retrospective single-center study	Tumor size (≤3, 3.1–5, 5.1–6.5, >6.5 cm) and number (1, 2–3, 4–5, >5)	AFP (≤20, 20.1–200, 200.1–1000, >1000 ng/mL)	-	Score 3–6 (transplantable): 3-year RFS: 87% 3-year OS: 79% Score 7–12 (non-transplantable): 3-year RFS: 31% 3-year OS: 38%
SMC criteria [51]	2007	Retrospective single-center study	Tumor size ≤5 cm	AFP level ≤400 ng/mL	-	Within criteria: 5-year DFS: 88.4% 5-year OS: 86.8% Outside criteria: 5-year DFS: 42.1% 5-year OS: 23.3%
Up-to-7 criteria [10]	2009	Retrospective multicenter study	Sum of the largest tumor size and number of lesions <7	-	-	5-year OS: 71.2% (beyond Milano and within up-to-7 criteria)
AFP-French model [11]	2012	Retrospective multicenter study (training + validation cohort)	Tumor size (≤3, 3–6, >6 cm) and tumor number (1–3, ≥4)	log_10_(AFP) Simplified version: AFP level (≤100, 100–1000, >1000 ng/mL)	-	Low-risk (score ≤2) 5-year recurrence rate: 13.4% 5-year OS: 69.9% High-risk (score >2) 5-year recurrence rate: 45.3% 5-year OS: 40.8%
AFP/TTD criteria [47]	2012	Retrospective multicenter study	Total tumor diameter ≤8 cm	AFP ≤ 400 ng/mL	-	Recurrence rate (43 months of FU): In criteria: 4.9% Outside criteria: 33.0% 5-year DFS similar compared to Milan criteria: 74.4% vs. 72.9%
TTV/AFP model [12]	2015	Prospective multicenter study	Total tumor volume <115 cm ^3^	AFP < 400 ng/mL	No macrovascular invasion; no extrahepatic disease	4-year DFS: 68.0% 4-year OS: 74.6% (beyond Milano and within TTV/AFP)
TRAIN score [44]	2016	Retrospective evaluation of prosepctively collected data, two centers (training + validation cohorts)	-	AFP slope ≥15 ng/mL/month	Radiological response to locoregional treatment (mRECIST) NLR ≥ 5 at liver transplant Length of waiting-time (months)	In criteria (score <1) 5-year ITT survival analysis: 67.5% 5-year recurrence rate: 8.9% Outside criteria (score ≥1) 5-year ITT survival analysis: 23.5% 5-year recurrence rate: 30.0%
Extended Toronto criteria [13]	2016	Prospective single-center study	Any size or number of tumors	-	No vascular invasion; no extrahepatic disease No cancer related symptoms (weight loss >10 kg and or ECOG ≥1 in 3 months) No poorly differentiated tumors	Beyond Milano and within ETC: 10-year risk of recurrence: 33% (vs. 15% for Milano in) 10-year survival: 50% (vs. 60% for Milano in)
Pre-MORAL score [45]	2017	Prospective single-center study	Largest tumor size >3 cm	Maximum AFP >200 ng/mL Preoperative NLR ≥5	-	5-year RFS: Low-risk group (score 0–2): 98.6% Medium-risk group (score 3–6): 69.8% High-risk group (score 7–10): 55.8% Very high-risk group (score >10): 0% (1-year RFS 17.9%)
EurHeCaLT transplant benefit model [55]	2017	Retrospective multicenter study	Single tumor >5 cm or ≤3 tumors ≤ 3 cm (Milano in) considered as a negative factor	AFP ≥ 1000 ng/mL considered as a negative factor	Considered as negative factors: MELD ≤13 CR or PD after locoregional treatment (mRECIST)	Transplant benefit: 3–4 negative factors: 0 months (no benefit) 2 negative factors: 20 months (small benefit) 1 negative factor: 40 months (moderate benefit) 0 negative factors: 60 months (large benefit)
HALT-HCC score [46]	2017	Retrospective single-center study	Hypotenuse between lesion number and lesion size (TBS)	ln(AFP)	MELD-Na	Risk equation: 1.27 × TBS + 1.85 × ln(AFP) + 0.26 × MELD-Na 5-year OS: Quartile 1: 78.7% Quartile 2: 74.5% Quartile 3: 71.8% Quartile 4: 61.5%
NYCA score [38]	2018	Retrospective evaluation of prosepctively collected data, multicenter	Maximum tumor size (0–3, 4–6, >6) and maximum tumor number (1, 2–3, ≥4)	AFP response (max to final)	-	5-year RFS: Low-risk (score 0–2): 90% Acceptable risk (score 3–6): 70% High-risk (score ≥7): 42%
Metroticket 2.0 model [14]	2018	Retrospective evaluation of prosepctively collected data, multicenter (training, internal validation and external validation set)	Tumor number and size of the largest tumor ^†^	AFP (<200, 200–400, 400–1000, >1000 ng/mL) ^†^	-	5-year RFS: within criteria 89.6% vs. beyond criteria 46.8% 5-year OS: within criteria 79.7% vs. beyond criteria 51.2% (with a tumor-specific survival of 93.5% within vs. 55.6% beyond)
Metroticket 2.0 + mRECIST criteria [56]	2020	Retrospective evaluation of prosepctively collected data, multicenter	Tumor number and size of the largest tumor	AFP (<200, 200–400, 400–1000, >1000 ng/mL)	Radiological response to neoadjuvant therapies (mRECIST criteria)	5-year HCC-related death: CR: 3.1% PR/SD: 9.6% PD: 13.4% In comparison to metroticket 2.0, the inclusion of radiological response resulted in reclassification of 9.4% of patients who died from HCC-related death within 5 years from LT
Metroticket 2.0 with LI-RADS criteria [57]	2021	Retrospective single-center study	Tumor number and size of the largest tumor (Tumor burden evaluated according to LI-RADS criteria)	log_10_(AFP)	-	Nodules identified with EASL non-invasive criteria: c-index = 0.72 (95% CI 0.64–0.80) [Ref] LR-5 and LR-TR-V nodules: c-index = 0.60 (95% CI 0.48–0.72) [*p* = 0.009] LR-4, LR-5 and LR-TR-V nodules: c-index = 0.60 (95% CI 0.48–0.72) [*p* = 0.007] LR-3, LR-4, LR-5 and LR-TR-V nodules: c-index = 0.65 (95% CI 0.54–0.76) [*p* = 0.08]

^†^ Criteria for transplantability: HCC with up-to-7 criteria if AFP <200 ng/mL; HCC within up-to-5 criteria if AFP 200–400 ng/mL; HCC within up-to-4 criteria if AFP 400–1000 ng/mL (considering as up-to-7, to 5 and to 4 the maximum allowed sum of size (cm) and number of tumors derived in any given HCC before transplantation, whether or not preceded by neoadjuvant therapies). Abbreviations: OS, overall survival; RFS, recurrence-free survival; AFP, alpha-fetoprotein; DFS, disease-free survival; TTD, total tumor diameter; TTV, total tumor volume; FU, follow-up; mRECIST, modified Response Evaluation Criteria in Solid Tumors; NLR, neutrophils-to-lymphocytes ratio; ETC, extended Toronto criteria; MELD, Model for End-stage Liver Disease; CR, complete response; PD, progressive disease; TBS, tumor burden score; LI-RADS, Liver Imaging Reporting and Data System; EASL, European Association for the Study of the Liver.

**Table 2:** Selected studies investigating the role of AFP in liver transplantation as a marker of prognosis and risk of recurrence.

Authors	Year	Design	Criteria	N. of Patients	AFP Details	Survival/Recurrence Risk ^†^
AFP in the selection of liver transplantation candidates						
Yang et al. [48]	2007	Retrospective single-center study	Seoul criteria	63	Last AFP: ≤20, 20.1–200, 200.1–1000, >1000 ng/mL	3-year OS: Score 3–6 (transplantable): 79% Score 7–12 (non-transplantable): 38% 3-year RFS: Score 3–6: 87% Score 7–12: 31%
Kwon et al. [51]	2007	Retrospective single-center study	SMC criteria	139	Last AFP ≤400 ng/mL	5-year OS: In criteria (tumor ≤5 cm and AFP ≤ 400 ng/mL): 86.8% Outside criteria: 23.3% 5-year DFS: In criteria: 88.4% Outside criteria: 42.1%
Ravaioli et al. [53]	2008	Prospective single-center study	Bologna criteria for downstaging	48	AFP remained <400 ng/mL during waiting time	Recurrence rate: 18.8% 3-year DFS: 71% 3-year OS: 72%
Vibert et al. [33]	2010	Retrospective single-center study	-	153	Preoperative AFP Progressors: >15 μg/L per month Non-progressors: ≤15 μg/L per month	5-year OS/RFS: Progressors: 54%/47% Non-progressors: 77%/74%
Merani et al. [36]	2011	Retrospective cohort study	-	6817	AFP_L_ >400 ng/mL downstaged to ≤400 ng/mL AFP_L_ >400 ng/mL failed to reduce to ≤400 ng/mL AFP_L_ stable at ≤400 ng/mL	Intention-to-treat OS at 3 years: AFP downstaged: 81% AFP not downstaged: 48% AFP stable at low levels: 74%
Duvoux et al. [11]	2012	Retrospective multicenter study (training + validation cohort)	AFP model	435	log_10_ (AFP_L_) Simplified version: Low-risk: AFP ≤ 1000 or 100–1000 ng/mL High-risk: AFP >1000 ng/mL	5-year recurrence rate/OS: Low-risk:13.4%/69.9% High-risk: 45.3%/40.8%
Lai et al. [47]	2012	Retrospective multicenter study	AFP-TTD criteria	158	Last AFP ≤400 ng/mL	Recurrence rate (median FU 43 months): In criteria (TTD ≤ 8 cm and AFP ≤ 400 ng/mL): 4.9% Outside criteria: 33.0%
Berry et al. [28]	2013	Retrospective cohort study	-	8659	AFP at transplant: ≤15 ng/mL 16–65 ng/mL 66–320 ng/mL >320 ng/mL	Compared to patients without HCC progressively increasing mortality risk: ≤15 ng/mL: aHR = 1.02 (95% CI 0.93–1.12) 16–65 ng/mL: aHR = 1.38 (95% CI 1.23–1.54) 66–320 ng/mL: aHR = 1.65 (95% CI 1.45–1.88) >320 ng/mL: aHR = 2.37 (95% CI 2.06–2.73)
Lai et al. [25]	2013	Prospective multicenter study	-	422	AFP progression >15 ng/mL/month	AFP slope was an independent risk factor for recurrence: HR = 5.4 (95% CI 2.8–10.1)
Vitale et al. [62]	2014	Retrospective cohort study	Italian transplant benefit model	4399	AFP: ≤100, 100–1000, >1000 ng/mL	Development of “HCC-MELD” score, an equation producing a numerical score that matches HCC patients with non-HCC patients in waiting list: 1.27 × MELD—0.51 × logAFP + 4.59
Toso et al. [12]	2015	Prospective multicenter study	TTV/AFP model	233	AFP_L_ ≤400 ng/mL	Milan in: 4-year DFS: 77.9% 4-year OS: 78.7% Milan out but TTV/AFP in: 4-year DFS: 68.0% 4-year OS: 74.6%
Lai et al. [44]	2016	Retrospective evaluation of prosepctively collected data, two centers (training + validation cohorts)	TRAIN score	Training set: 179 Validation set: 110	AFP slope ≥15 ng/mL/months	ITT 5-year survival in/outside criteria: Training set: 67.5%/23.5% Validation set: 66.7%/20.7% ITT 5-year recurrence rate in/outside criteria: Training set: 8.9%/30.0% Validation set: 13.8%/100.0%
Sapisochin et al. [13]	2016	Prospective single-center study	Extended Toronto criteria	588	AFP_L_ <500 ng/mL	1-, 3- and 5-year patient survival: <500 ng/mL: 60%, 43% and 37% ≥500 ng/mL: 88%, 73% and 64%
Halazun et al. [45]	2017	Prospective single-center study	Pre-MORAL score	339	Maximum AFP from HCC diagnosis to LT >200 ng/mL	5-year RFS: Low-risk (score 0–2): 98.6% Medium-risk (score 3–6): 69.8% High-risk (score 7–10): 55.8% Very high-risk group (score >10): 0% (1-year RFS 17.9%)
Lai et al. [55]	2017	Retrospective multicenter study	EurHeCaLT transplant benefit model	2103	Last AFP ≥1000 ng/mL	ITT transplant benefit: ≥1000 ng/mL: 6.8 months <1000 ng/mL: 25.4 months
Halazun et al. [38]	2018	Retrospective evaluation of prosepctively collected data, multicenter	NYCA score	1450	AFP response (max AFP to final AFP)	5-year RFS: Low-risk (score 0–2): 90% Acceptable risk (score 3–6): 70% High-risk (score ≥7): 42%
Mazzaferro et al. [14]	2018	Retrospective evaluation of prosepctively collected data, multicenter (training, internal validation and external validation set)	Metroticket 2.0 model	Training set: 1081 Validation set: 341	Pre-transplant AFP: <200, 200–400 ng/mL, 400–1000 and >1000 ng/mL	5-year RFS: within criteria 89.6% vs. beyond criteria 46.8% 5-year OS: within criteria 79.7% vs. beyond criteria 51.2% (with a tumor-specific survival of 93.5% within vs. 55.6% beyond)
Mehta et al. [37]	2019	Retrospective cohort study	-	407	Pre-LT AFP >1000 ng/mL: Persistently >1000 ng/mL Decreased to 100–499 ng/mL Decreased to ≤100 ng/mL	5-year recurrence probability/OS: Persistently >1000 ng/mL: 35.0%/48.8% Decreased to 100–499 ng/mL: 13.3%/67.0% Decreased to ≤100 ng/mL: 7.2%/88.4%
AFP in the prediction of risk of tumor recurrence						
Han et al. [32]	2007	Retrospective single-center study	-	48	Preoperative AFP slope >50 μg/L per month	One-year RFS: >50 μg/L: 40% ≤20 μg/L: 90%
Dumitra et al. [35]	2013	Retrospective single-center study	-	92	Pre-operative AFP slope >0.1 μg/L/day	Pre-operative AFP slope independently associated with: Post-LT recurrence: OR = 3.98 (95% CI 1.01–15.81)
Hameed et al. [54]	2014	Retrospective single-center study	-	211	Pretransplant AFP <1000 ng/mL	5-year RFS: ≤1000 ng/mL: 80.3% >1000 ng/mL: 52.7%
Grat et al. [50]	2016	Retrospective single-center study	-	146	AFP persistently <100 ng/mL AFP >100 ng/mL reduced to <100 ng/mL AFP rising to >100 ng/mL AFP persistently >100 ng/mL	5-year RFS: Persistently <100 ng/mL: 97.3% From >100 ng/mL to <100 ng/mL: 100% From <100 ng/mL to >100 ng/mL: 75% Persistently >100 ng/mL: 38.4%
Piñero et al. [63]	2016	Retrospective multicenter study	-	323	AFP_L_: ≤100 ng/mL 101–1000 ng/mL >1000 ng/mL	5-year incidence of recurrence: ≤100 ng/mL: 11.1% 101–1000 ng/mL: 19.7% >1000 ng/mL: 38.9%
Agopian et al. [39]	2017	Retrospective cohort study	-	3601	AFP change/response after LRT	Risk of HCC recurrence: Pre-LT AFP = max, but <20 ng/mL: HR = 1 Pre-LT AFP < max, normalized <20 ng/mL: HR = 0.9 (*p* = 0.6) Pre-LT AFP < max, improving but still >20 ng/mL: HR = 2.0 (*p* < 0.001) Pre-LT AFP = max and >20 ng/mL: HR = 3.1 (*p* < 0.001)
Notarpaolo et al. [64]	2017	Retrospective multicenter study	-	574	Last AFP before LT: ≤100 ng/mL 100–1000 ng/mL >1000 ng/mL	5-year risk of recurrence: ≤100 ng/mL: 13.0% 100–1000 ng/mL: 34.9% >1000 ng/mL: 75.0%
Mehta et al. [29]	2017	Retrospective multicenter study (development and validation cohort)	RETREAT score	1061 (721 in development cohort and 341 in validation cohort)	Pre-operative AFP: 0–20 ng/mL 21–99 ng/mL 100–999 ng/mL ≥1000 ng/mL	Increase of the 5-year recurrence risk from 2.9% of patients with score 0 to 75.2% of patients with score ≥5
Mehta et al. [30]	2018	Retrospective cohort study	RETREAT score	3276	Pre-operative AFP: 0–20 ng/mL 21–99 ng/mL 100–999 ng/mL ≥1000 ng/mL	3-year recurrence risk: Score 0: 1.6% Score 1: 5.0% Score 2: 5.6% Score 3: 8.4% Score 4: 20.3% Score ≥5: 29.0%
Giard et al. [34]	2018	Retrospective single-center study	-	336	Pre-operative AFP slope >7.5 ng/mL per month	AFP slope >7.5 ng/mL per month was independently associated with: HCC recurrence: HR = 3.0 (95% CI 1.1–8.1) Microvascular invasion: OR = 6.8 (95% CI 1.6–28.7)
Di Norcia et al. [40]	2020	Retrospective cohort study	-	3439	logAFP immediate pre-transplant	In patients without complete pathologic response after LRT, AFP independently predicted recurrence: HR = 1.45 (95% CI 1.29–1.64) 5-year recurrence risk: Low-risk group: 5.8% Moderate risk group: 24% High-risk group: 69%

† In the Table are presented data regarding AFP in the selection of liver transplantation candidates or in prognosis prediction. Other disease-related criteria that may be included in the scoring systems are not presented in the Table for the sake of simplicity. Please refer to Table 1 or to the original publication for additional details. Abbreviations: AFP, alpha-fetoprotein; OS, overall survival; RFS, recurrence-free survival; DFS, disease-free survival; TTV, total tumor volume; AFPL, AFP at listing; TTD, total tumor diameter; FU, follow-up; ITT, intention-to-treat; LT, liver transplantation; aHR, adjusted hazard ratio; LRT, locoregional therapies.

**Table 3:** Proposed post-transplant scoring systems.

Model Name	Year	Design	Tumor Burden	Biomarker	Other Criteria	Performance
Parfitt et al. [65,70]	2007	Retrospective single-center study	Tumor size ≥3 cm	-	Microvascular invasion Satellitosis Giant/bizarre cells >25% visible at low power	Recurrence in: Low-risk (score 0–4): 4.3% Intermediate-risk (score 7–7.5): 28.5% High-risk (score 10–14): 50.0% At the cut-off of 3.5: AUROC = 0.8, sensitivity 80%, specificity 79%
		Retrospective single-center study				
Decaens et al. [71]	2011	Retrospective single-center study (training and validation cohorts)	Number of nodules (1, 2–3, ≥4) and maximal diameter of the largest nodule (≤2, 2–3, 3–5, >5 cm)	-	Tumor differentiation (well, moderate, poor)	Training cohort: AUROC = 0.65 (95% CI 0.59–0.71) 5-year tumor free survival: 60.2% with score <4 and 36.4% with score ≥4 (*p* < 0.0001) Validation cohort: AUROC = 0.63 (95% CI 0.50–0.76) 5-year tumor free survival: 82.8% with score <4 and 50.0% with score ≥4 (*p* < 0.0001)
UCLA nomogram [42]	2015	Retrospective evaluation of prospectively collected data, single-center	Within Milano/downstaged to Milano vs. Milano out Maximal radiological tumor diameter	AFP NLR Total cholesterol	Microvascular invasion Tumor grade	C statistic of 0.85 (95% CI 0.82–0.89) Nomogram predicting 1-, 3- and 5-year recurrence risk for any individual patient with HCC
Post-Moral [45]	2017	Retrospective evaluation of prospectively collected data, single-center	On explant pathology: Largest size >3 cm Tumor number >3	-	Vascular invasion Tumor grade	5-year RFS: Low-risk group (score 0–2): 97.4% Medium-risk group (score 3–6): 75.1% High-risk group (score 7–10): 49.9% Very high-risk group (score >10): 22.1%
RETREAT score [30]	2018	Retrospective cohort study	Explant largest viable tumor diameter + number of viable tumor (0, 1–4.9, 5–9.9, >10)	AFP at LT (0–20, 21–99, 100–999, >1000 ng/mL)	Presence of microvascular invasion	3-year recurrence risk: Score 0: 1.6% Score 1: 5.0% Score 2: 5.6% Score 3: 8.4% Score 4: 20.3% Score ≥5: 29.0%

Abbreviations: AUROC, area under Receiver Operating Characteristic (ROC) curve; AFP, alpha-fetoprotein; NLR, neutrophils-to-lymphocytes ratio; RFS, recurrence-free survival; LT, liver transplantation.

**Table 4:** Studies investigating the pattern of HCC recurrence.

Study	Year	Patients with Recurrence	Time to Recurrence (Months)	Hepatic Recurrence	Extrahepatic Recurrence	Multiorgan Recurrence	Survival after Recurrence	MINORS Score [88]
Regalia et al. [89]	1998	21/132 (15.9%)	7.8 (range, 1–25)	4 (3%)	17 (12.9%) 4 lung, 3 bones, 1 peritoneum, 1 subcutaneous	15 (11.4%)	OS rates at 1-, 2-, 3- and 4-year: 62%, 43%, 29% and 23%	9/16
Schlitt et al. [90]	1999	39/69 (56.5%)	14.5	24 (34.8%)	30 (43.5%) 22 lung, 7 bone, 5 adrenal glands, 4 lymph nodes	19 (27.5%)	No antitumor therapy: median 172 days Non-surgical therapy: median 245 days	9/16
Roayaie et al. [78]	2004	57/311 (18.3%)	12.3 (range, 1.5–60.3)	9 (15.9%)	30 (52.6%)	18 (31.6%)	8.7 months	9/16
Escartin et al. [17]	2007	28/184 (15.2%)	Early recurrence (≤12 months): 9 (32.1%) Late recurrence (>12 months): 19 (67.9%)	7 (25%)	21 (75%) 7 lung, 5 bones, 2 lymph nodes, 2 skin, 2 adrenal glands, 2 peritoneum, 1 CNS	11 (39.3%)	7.0 months	7/16
Shin et al. [81]	2010	28/138 (20.3%)	7.9 (range, 1.8–38.2)	14 (10.1%)	24 (17.4%) 10 lung, 8 bones, 3 lymph nodes, 2 peritoneum, 1 adrenal glands	7 (5.1%)	11.7 months (range, 1.1–50.7)	7/10
Cescon et al. [80]	2010	34/283 (12%)	12.0 (range, 1–118) In 2 cases recurrence 7 and 9 years after LT	3 (8.8%)	7 (20.6%) 3 lung, 2 peritoneum, 2 bones	24 (70.6%)	-	11/16
Taketomi et al. [91]	2010	17/101 (16.8%)	12.9 (range, 1.7–60.2)	-	-	-	12.0 months (2.2–31.1)	9/16
Valdivieso et al. [19]	2010	23/182 (12.6%)	23.4 (range, 2–93)	2 (8.7%	16 (69.5%)	5 (21.7%)	Patients with R-0 surgery after recurrence: 33.2 ± 21.5 months Other patients: 11.9 ± 6.9 months	7/16
Kornberg et al. [73]	2010	16/60 (26.7%)	23 (range, 4–58)	4 (25%)	12 (75%) 5 lung, 4 bones, 1 CNS, 1 adrenal gland, 1 peritoneum	-	10.5 months (range, 1–136)	8/16
Sharma et al. [74]	2012	17/94 (18%)	25.2	6 (35.3%)	6 (35.3%) 3 adrenal glands, 1 lung, 2 abdominal mass	11 (64.7%)	-	9/16
Roh et al. [92]	2014	63/458 (13.8%)	12.9 (range, 0.4–82.4)	14 (22%)	16 (26%) 10 lung, 2 bone, 2 adrenal gland, 1 peritoneum, 1 lymph node	33 (52%)	12 months (range, 1.1–86.6)	8/16
Sapisochin et al. [72]	2015	121/780 (15.5%)	14 (range, 1.4–98.2)	16 (13.2%)	63 (52.1%)	42 (34.7%)	12.2 months (range, 0.1–112.5)	12/16
Mehta et al. [29]	2017	84/721 (11.6%)	13.0 (IQR, 5.4–26.7)	22 (26.2%)	84 (100%) 37 lung, 25 bones, 22 peritoneum	21 (25%)	-	10
Fernandez-Sevilla et al. [75]	2017	70/493 (14.2%)	17 (range, 10–35)	2 (2.8%)	51 (72.9%)	17 (24.3%)	19 months (35 in resected patients vs. 15 in non-resected patients)	14/16
Alshahrani et al. [77]	2018	232/1486 (15.6%)	Recurrence <1 year: 117 Recurrence 2–5 years: 93 Recurrence >5 years: 22	72 (31.0%)	134 (57.8%)	31 (13.4%)	Post recurrence survival (months): Recurrence <1 year: 10.2 Recurrence 2–5 years: 23.8 Recurrence >5 years: 37.0	8/16

Abbreviations: LT, liver transplantation; CNS, central nervous system; IQR, interquartile range.
